# Comparison of 1- and 2-year screening intervals for women undergoing screening mammography

**DOI:** 10.1038/sj.bjc.6602393

**Published:** 2005-02-15

**Authors:** E S Wai, Y D'yachkova, I A Olivotto, S Tyldesley, N Phillips, L J Warren, A J Coldman

**Affiliations:** 1 Radiation Therapy Program, BC Cancer Agency, Vancouver Island Centre, 2410 Lee Ave, Victoria, BC, Canada V8R 6V5; 2 Population and Preventive Oncology Program, BC Cancer Agency, 8th Floor, 686 West Broadway, Vancouver, BC, Canada V5Z 1G1; 3 Radiation Therapy Program, BC Cancer Agency,Vancouver Centre, 600 West 10th Ave, Vancouver, BC, Canada V5Z 4E6; 4 Screening Mammography Program of BC, 8th Floor, 686 West Broadway, Vancouver, BC, Canada V5Z 1G1

**Keywords:** mammography, screening frequency, screening interval, breast cancer survival

## Abstract

We compared the long-term impact of 1- and 2-year screening mammography intervals using prognostic, screening, and outcome information for women aged 50–74 years obtained from the Screening Mammography Program of British Columbia in two time periods, prior to 1997 (policy of annual mammography) and after 1997 (biennial mammography). Survival was estimated for both periods using a prognostic model and the expected rate of interval and screen-detected cancers. The likelihood of a screen-detected cancer with annual screening was 2.32 per thousand screens and with biennial screening was 3.32 per thousand screens. The prognostic profile of screen-detected cancers was better than that of interval cancers. Among both screen-detected and interval cancers, the prognostic profiles with annual and biennial screening were similar. The estimated breast cancer-specific survival rates for women undergoing annual and biennial screening mammography were 95.2 and 94.6% at 5 years, and 90.4 and 89.2% at 10 years, respectively. Annual compared to biennial mammography was associated with a 1.2% increase in the estimated 10-year breast cancer-specific survival for women aged 50–74 years, diagnosed with invasive breast cancer after screening programme attendance.

Screening mammography reduces breast cancer mortality (Chu *et al*, 1988; Chu and Connor, 1991; Nystrom *et al*, 1993; Kerlikowske *et al*, 1995; Bjurstam *et al*, 1997; Alexander *et al*, 1999; Tabar *et al*, 2000; Black *et al*, 2002; Nystrom *et al*, 2002). A meta-analysis of the randomised screening trials found equivalent mortality reductions (23%) from approximate annual screening compared to screening every 18–33 months (Kerlikowske *et al*, 1995). The only randomised trial to address the frequency of recall between screens found that modelled 10- and 15-year breast cancer survival was not significantly different for women undergoing screening mammograms every year *vs* every 3 years (Breast Screening Frequency Trial Group, 2002).

In British Columbia (BC), the third largest province in Canada with a population of 4.2 million, the population-based Screening Mammography Program (SMPBC) has provided bilateral two-view mammograms to the asymptomatic female residents of BC, aged 40 and older, free of direct payment since 1988 (Olivotto *et al*, 2000). In 1997, the SMPBC increased its recommended screening interval for women aged 50–74 years from yearly to every 2 years.

We have examined the effect of this change in screening frequency policy. As follow-up time since the policy change is too short to demonstrate a difference in mortality, the impact on breast cancer-specific survival was estimated by applying the observed differences in the prognostic profile of cancers diagnosed in women screened at different intervals to a prognostic prediction model.

## MATERIALS AND METHODS

This retrospective study modelled 10-year survival outcomes for women, aged 50–74 years, screened annually and biennially through the SMPBC. Two time periods were compared, prior to 1997 (‘annual’ screening group) and after 1997 (‘biennial’ screening group).

### Data sources

Electronic records for participants in the SMPBC were collected from the SMPBC database and the BC Cancer Registry, both of which are administered through the BC Cancer Agency. The SMPBC database contains demographic, prognostic, and outcome information on all women screened through the programme and maintains active follow-up to ascertain final pathology results for women with abnormal screening mammograms. It is linked every 3 months with the BC Cancer Registry to identify all breast cancers (screen-detected or otherwise) in screened women. The BC Cancer Registry includes notifications of all cancer reported within the province on either pathology reports or death certificates. The capture rate for new cancers was 94% for the year 1999. Vital status, date, and cause of death are obtained secondary to regular electronic linkage with the Vital Statistics Agency, Ministry of Health, Government of British Columbia.

For this study, a cancer was defined as an invasive epithelial cancer of any histology or ductal carcinoma *in situ* (DCIS) (i.e. sarcoma, lymphomas, phyllodes tumours, and lobular carcinoma *in situ* lesions were excluded). Cancers were classified as screen-detected if an abnormal screen led to a diagnostic sequence that resulted in the detection of a breast cancer. All other cancers were classified as interval cancers. Subjects were assumed to be at risk of developing an interval cancer from the time of one screen until the time of the first of either the subsequent screen, a diagnosis of an interval cancer, death, or the date of December 31, 2001.

### ‘Prognostic’ and ‘screening’ data samples

Two overlapping sets of data were extracted. The ‘prognostic sample’ consisted of all cases of unilateral breast cancer diagnosed prior to January 1, 2002 in women ages 50–74 years, who had attended the SMPBC. Prognostic and outcome information for these cases was collected from the SMPBC database.

The ‘screening sample’ consisted of all women who received at least one screen at ages 50–74 years, between 1988 and December 31, 2001. Each woman's screening history was subdivided into screening intervals, which started immediately after a screen and terminated with the first of: another screen, death, diagnosis of cancer or December 31, 2001. Since this was an analysis of recall frequency, women diagnosed with cancer at their first screen were excluded. Only screening intervals in which the woman was aged 50–74 years at the beginning were used.

### ‘Annual’ and ‘biennial’ screening frequency

Screening mammograms performed 10–14 months after the prior screen and prior to January 1, 1997 were defined as ‘annual’ screens. Screening mammograms performed 20–28 months after the prior screen and after December 31, 1997 were defined as ‘biennial’ screens. These time intervals were created because most women did not return exactly 12 or 24 months after a previous screen. These intervals of 10–14 months, pre-1997, and 20–28 months, post-1997, were chosen to maintain a period clinically similar to the 1- and 2-year screening intervals of primary interest, and to ensure a similar proportion of women returned for re-screening (approximately 50% for each of the time periods studied). Each screening interval was analysed independently, so that women who had screens prior to and after 1997 could have contributed screening intervals both to the annual and biennial screening groups. As the SMPBC policy change from annual to biennial screening was implemented in July 1997, the information from the entire transition year of 1997 was not used.

### Analytic schema/statistical methods

The following analytic approach was used:

1. *Prognostic model*: A Cox proportional hazards model was developed to predict breast cancer-specific survival based on methods published by Tabar *et al* (1995) and Haybittle *et al* (1982), using the ‘prognostic sample’ described above. Age, tumour size (⩽9, 10–14, 15–19, 20–29, 30–49, 50+ mm, unknown), nodal status (negative, positive, unknown) and histologic grade (well, moderately, or poorly differentiated and unknown) were examined as potential prognostic factors. As it was assumed that the 10-year breast cancer survival from a diagnosis of DCIS was 100% (Fisher *et al*, 1999; Julien *et al*, 2000), DCIS was not included in the model. Tests of significance were based on the partial likelihood method. The underlying hazard was estimated by the Breslow estimator. Partial likelihood methods were used to generate approximate confidence interval (Table 1).
2. *Prognostic distribution*: The ‘screening sample’ described above was used to identify cases of cancer falling into the following four groups: (i) interval cases occurring up to 12 months after the previous screen in either the ‘annual’ or ‘biennial’ screening group, (ii) screen-detected cases occurring 10–14 months after the previous screen among those in the ‘annual’ screening group; and for those in the ‘biennial’ screening group, (iii) interval cases occurring 12–24 months after the previous screen, and (iv) screen-detected cases occurring 20–28 months after the previous screen (Table 2). For each case in a group, the prognostic factors at presentation were used to predict that woman's survival using the pro-gnostic model. These were then averaged across the members of each group to provide an average predicted survival for the particular group at 5 and 10 years (Table 3).
3. *Weighting*: The distribution of screens (Table 4) and number of cancer cases from the ‘screening sample’ described above were then used to calculate the rates of cancer expected in each of the above four groups (Table 5) Rates of interval cancers were estimated using the Kaplan–Meier method and rates of screen-detected cancers were calculated using the observed proportions for the groups described earlier. These were then used to create weights reflecting the distribution of cancer cases by screening the frequency group, detection method, and time of detection in the above four groups.
4. *Prediction*: The weights (from (3) above) were then applied to the average group survival rates (from (2) above) to provide the predicted 5- and 10-year survival rates.

## RESULTS

**Figure 1:**
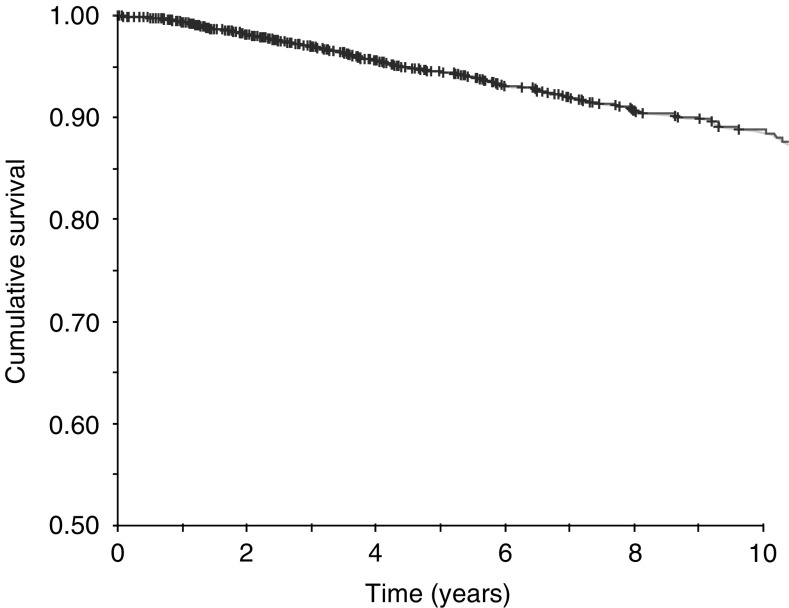
Breast cancer-specific survival rate for 5844 women ever attending SMPBC and subsequently diagnosed with unilateral invasive breast cancer, 1988–2002.

Following 897 216 screens provided to women aged 50–74 years between 1988 and 2001 at the SMPBC, 5844 cases of invasive, unilateral breast cancer were diagnosed. The distribution of tumour size, grade, and lymph node status at diagnosis is given in Table 1. The median follow-up from date of diagnosis was 42 months. The overall survival curve for these cases is shown in Figure 1.

In the Cox analysis of the prognostic sample, age was not significantly associated with breast cancer-specific mortality (*P*=0.095), but size, grade and lymph node status were (Table 1). The category ‘unknown’ was associated with a similarly poor prognosis for all variables considered and was combined into a composite category of ‘unknown for any variable’, which was a strongly negative prognostic factor. The hazard ratios for breast cancer mortality for the relevant prognostic factors are provided in Table 1. The observed distribution of prognostic factors for the 2441 cancer cases in the screening sample is shown in Table 2. The prognostic profile of screen-detected cancers was better than that of interval cancers, while the profile of screen-detected cancers was similar for those found at the ‘annual’ or the ‘biennial’ screen. This was also true for interval cancers diagnosed between 0–12 and 12–24 months. This similarity in prognostic factors translated into minimal differences in estimated 5- and 10-year breast cancer-specific survival rates for each type of tumour, as calculated using the prognostic model developed from the ‘prognostic sample’ and prognostic factors in the ‘screening sample’ (Table 3).

The distribution of screening intervals is shown in Table 4, the mean interval for those screened prior to 1997 being 12.4 months compared to 24.1 months for those screened after 1997. Approximately two-thirds of the screening intervals prior to 1997 were 10–14 months, whereas only 20% of those after 1997 were of that length, coincident with the change in SMPBC policy. Approximately half the screening intervals after 1997 were 20–28 months. The age distribution of women aged 50–74 years was similar for the two periods, with means of 61.0 years prior to 1997 and 61.2 years after 1997.

Of those included in the screening sample, the likelihood of having a screen-detected cancer diagnosed with an ‘annual’ screening interval prior to 1997 was 2.32 per thousand screens (438 out of 188 709) and for women with a ‘biennial’ screening interval after 1997 was 3.32 (692 out of 208 723). The rate of interval cancer by time since last screen was similar over the first 12 months after a screen before 1997 (0.71 per 1000) and after 1997 (0.80 per 1000). The cumulative rate of interval cancers by 24 months was 2.33 per 1000 after 1997. The resulting estimated numbers of screen-detected and interval-invasive cancers per 100 000 women screened and per 100 000 person-years, respectively, for a period of 2 years, either ‘annually’ before 1997 or ‘biennially’ after 1997 are given in Table 5. The overall numbers of invasive and *in situ* cancers predicted over the 24 months were very similar (716 *vs* 713) for the two screening frequencies, but more tumours were *in situ* in the post-1997 period.

Based on the distribution of screen-detected and interval cancers for the two screening strategies, the estimated breast cancer-specific survival rates for women aged 50–74 years undergoing annual as compared to biennial screening mammography were 95.2 and 94.6%, respectively, at 5 years, and 90.4 and 89.2%, respectively, at 10 years (Table 6). The absolute difference in estimated breast cancer survival between the annual and biennial screening strategies were 0.6% at 5 years and 1.2% at 10 years, corresponding to a relative risk of 0.89 in favour of annual screening, assuming 100% compliance.

**Figure 2:**
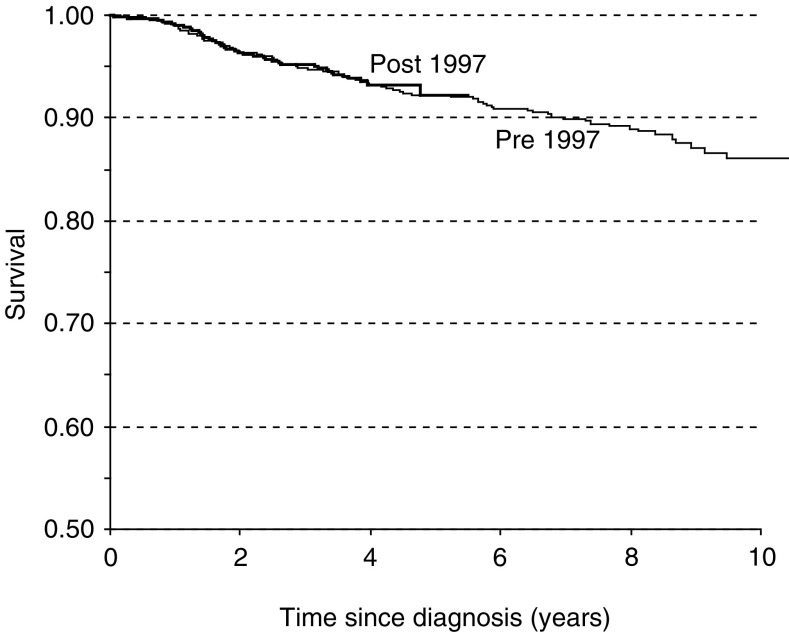
Observed survival curves for women aged 50–79 at screening and diagnosed with breast cancer prior to 1997 (annual screening period) or after 1997 (biennial screening period). Cases prior to 1997 were either interval cases ⩽12 months or screen-detected at 10–14 months; those diagnosed after 1997 were either interval cases ⩽24 months or screen-detected at 20–28 months.

Figure 2 shows the Kaplan–Meier survival curves for all women aged 50–79 attending the SMPBC, diagnosed with invasive, unilateral breast cancer prior to and after 1997. There was no difference in observed survival for patients diagnosed prior to or after the policy change of annual to biennial screening for women diagnosed with breast cancer after SMPBC attendance.

## DISCUSSION

This large, retrospective, population-based study of women aged 50–74 has found that annual screening mammography was associated with a higher proportion of screen-detected cancers than was biennial screening. The distribution of cancers and prognostic profiles translated into small absolute differences in modelled 5- and 10-year breast cancer-specific survival in a hypothetical cohort of 100 000 screens performed either annually or biennially (0.6 and 1.2%, respectively), in favour of the annually screened group. In keeping with this, there was no difference in observed survival in women diagnosed with ipsilateral, invasive breast cancer undergoing screening mammography during the time of an annual or biennial screening policy.

The predicted differences in 10-year breast cancer survival were similar to the findings of the only randomised trial addressing this issue, the United Kingdom Co-ordinating Committee on Cancer Research Randomised Trial performed (Breast Screening Frequency Trial Group, 2002). That study randomised women, aged 50–62 years, with a normal prevalent screen in the UK National Health Services (NHS) Breast Screening Programme, to a conventional incident screen after an interval of 3 years (control arm) or to three annual screenings (study arm).

The BSFTG randomised trial found that the incidence of breast cancers was 2.44 per thousand per annum in the study arm and 2.15 in the control arm, similar to the estimates from our study. Also, 71% of the cancers were screen-detected in the annual recall (study) arm compared to 50% in the 3-year recall (control) arm, similar to the rates, 76% in the annual and 58% in biennial screening cohorts found in this study. As expected, the shorter screening interval was associated with a higher proportion of screen-detected cancers, compared to interval cancers, but this translated to only a small absolute difference in the estimated 10-year breast cancer-specific survival: approximately 2% in the randomised trial and 1% in the current study.

In a meta-analysis of data from the randomised trials, there was no difference in the reduction in breast cancer mortality among women aged 50–74 with planned screening intervals of less than 18 months compared to those with longer intervals of 18–33 months (Kerlikowske *et al*, 1995).

Computer modelling simulations suggest that breast cancer mortality reductions improve with shorter screening intervals, with improvements as much as from 26%, for mammography performed every 5 years, to 66%, annual mammography (Szeto and Devlin, 1996; Michaelson *et al*, 1999, 2000; Fett, 2001). In contrast, Jansen *et al* used modelling to show that shorter screening intervals may be associated with greater detection of ‘excess’ tumours that would not have been detected before the subject died of other causes, and that the overall benefit to the population with respect to breast cancer mortality was greater when a larger proportion of the population were screened at a lower frequency compared to a smaller proportion screened more frequently (Jansen and Zoetelief, 1997).

Retrospective studies comparing interval and screen-detected cancers have shown that interval cancers are similar to cancers in unscreened women, and that screen-detected cancers generally have better prognostic indicators, with smaller tumours, fewer node-positive tumours, and a larger proportion of *in situ* tumours (Burrell *et al*, 1996; Porter *et al*, 1999). The current study confirms these observations, but of note is the similarity of the prognostic profile of interval cancers for those screened annually and that of interval cancers in those screened biennially, with the same being true for screen-detected cancers.

Retrospective cohort studies that examined prognostic differences of tumours detected in women who self-selected different screening frequencies have generally shown that those screened less frequently have tumours with worse prognostic profiles, including larger tumour size, more lymph node metastases, and fewer cases of isolated in-situ disease (Gabriel *et al*, 1997; Field *et al*, 1998; Carlson *et al*, 1999; Hunt *et al*, 1999). It has been shown that the higher absolute rate of false-positive screens increased with more frequent screening (Elmore *et al*, 1998).

A limitation of the current study is that, during the study interval (1995–2002), there could have been improvements in the technical aspects of imaging or in radiologist experience that may have affected detection rates (Kan *et al*, 2000; Feig, 2002; Haus, 2002; Taplin *et al*, 2002), although SMPBC data suggest that improved detection is unlikely to explain our observations. Within the SMPBC, women, aged 40–49, were recalled for screening annually both before and after 1997 and comparison of these periods showed little difference, with the cancer detection rate increasing only 0.1 per 1000 for both first and subsequent screens after 1997 (SMPBC unpublished data, December 2003). Another limitation was the incomplete enforcement of the change in screening policy with some women not following the re-screening recommendations in each period. This necessitated a wider range of duration between screens in the era after 1997 to create comparably sized groups for analysis purposes.

In spite of these limitations, this study provides useful information about different screening intervals based on actual data from a large, population-based screening programme that instituted a policy change, in the modern era of two-view, film-screen mammography. Complete and accurate outcome data were obtained through direct linkage to the provincial cancer registry. The two comparison groups were selected to minimise selection bias. The predicted survival was based on a prognostic model that used cases over the whole period to avoid the survival effect of changes in treatment between the different periods. Thus, the predicted survival was a ‘blended’ rate of the survival in each period and was applied uniformly to everyone in the study regardless of her date of diagnosis.

In conclusion, this study has shown that annual screening mammography minimally improved the estimated breast cancer survival rates for women aged 50–74 years, as compared to biennial screening at 5 and 10 years, with an absolute projected survival difference of 0.6% at 5 years and 1.2% at 10 years for those women diagnosed with breast cancer.

## Figures and Tables

**Table 1:** Distribution of tumour prognostic factors and corresponding adjusted hazard ratio for breast cancer mortality for 5844 invasive breast cancers diagnosed 1988–2001 in women undergoing screening mammograms through SMPBC (prognostic sample)

**Prognostic factor**	***N* (%)**	**Hazard ratio**	**(95% CI)**
*Tumour size (mm)*			
1–9	1163 (20)	1.0	—
10–14	1478 (25)	1.31	(1.20, 1.43)
15–19	1210 (21)	1.72	(1.45, 2.04)
20–29	1095 (19)	2.25	(1.74, 2.92)
30–49	480 (8)	2.95	(2.09, 4.16)
50+	224 (4)	3.87	(2.51, 5.95)
Unknown	194 (3)	3.36	(2.43, 4.66)

*Lymph node status*			
Negative	3608 (62)	1.0	—
Positive	1374 (23)	3.82	(2.91, 5.01)
Unknown	862 (15)	3.36	(2.43, 4.66)

*Histologic grade*			
Well differentiated	1583 (27)	1.0	—
Moderately differentiated	2074 (36)	1.04	(0.71, 1.54)
Poorly differentiated	1295 (22)	3.42	(2.42, 4.83)
Unkown	892 (15)	3.36	(2.43, 4.66)

CI=confidence interval.

**Table 2:** Distribution of prognostic factors among interval and screen-detected cancers in the screening sample

	**Interval cancer (%)**		**Screen-detected cancer (%)**	
**Factor**	**⩽12 months**	**12–24 months^a^**	**10–14 months^b^**	**20–28 months^c^**
Total number of cases (*n*)	656	655	438	692

*Tumour size (mm)*				
1–9	11	13	25	28
10–14	19	19	33	28
15–19	20	20	20	22
20–29	26	27	12	14
30–49	11	13	6	5
50+	8	7	2	1
Unknown	5	3	2	2

*Lymph node status*				
Negative	54	53	67	71
Positive	32	32	15	22
Unknown	14	14	18	7

*Histologic grade*				
Well	21	21	24	34
Mod	32	32	47	32
Poor	31	31	22	14
Unknown	17	16	7	20

*Unknown (size, node, or grade)*				
None	25	26	22	26
Any	75	74	78	74

a Including interval cancers diagnosed 20–28 months after a screen prior to January 1, 1997.

b Prior to January 1, 1997.

c After December 31, 1997.

**Table 3:** Estimated 5- and 10-year breast cancer specific-survival rates by type of cancer and time from most recent mammogram to diagnosis

	**Estimated 5-year survival rate (%)**	**Estimated 10-year survival rate (%)**
Screen-detected cancer at 10–14 months	96.0	91.8
Screen-detected cancer at 20–28 months	96.1	91.9

Interval cancer ⩽12 months	92.7	85.7
Interval cancer 12–24 months	92.5	85.3

**Table 4:** Distribution of screening intervals for women, age 50–74 years at time of an initial screen, who were re-screened prior to and after 1997

**Date of screen**	**Before 1997 (%)**	**After 1997 (%)**
*Length of interval (months)*		
0–9	1459 (0.5)	38 (0.0)
10–14 ‘annual’	188 709 (68)	80 767 (20)
14–19	49 585 (18)	53 908 (13)
20–28 ‘biennial’	24 940 (9)	208 723 (52)
28+	14 459 (5)	58 647 (15)

Total	279 152	402 083

**Table 5:** Observed distribution of invasive cancers by screening frequency for women, age 50–74 years, screened over 2 years either annually or biennially

**Mode of detection**	**Number of cases observed**	**Number at risk^a^**	**Observed rate per 100 000**	**Weighting of cancers by mode of detection (%)**
*Annual screening prior to 1997*				
Screen detected at 10–14 months	438	188 709	232	76.6
Interval cancers ⩽12 months	305	428 574	71	23.4

*Biennial screening after 1997*				
Screen detected at 20–28 months	692	208 723	332	58.8
Interval cancers ⩽12 months	351	439 550	80	14.1
Interval cancers 12–24 months	384	250 979	153	27.1

a For screen-detected cancers, unit is the number of screens; for interval cancers, unit is person-years.

**Table 6:** Predicted 5- and 10-year survival rates by frequency of screening^a^

**Screening frequency**	**Predicted 5-year survival rate (%)**	**Predicted 10-year survival rate (%)**
‘Annual’	95.2	90.4
‘Biennial’	94.6	89.2

a Predicted breast cancer-specific survival rates based on expected prognostic profile and distribution of interval and screen-detected cancers.
